# Supplementary material for: Cooperative molecular interaction networks govern PARP1 inhibitor selectivity and binding affinity
Source: PLoS Comput Biol. 2026 May 26;22(5):e1014296. doi: 10.1371/journal.pcbi.1014296 (PMC13241013; doi:10.1371/journal.pcbi.1014296)
Supplement: S1 Appendix — Potential of mean force (PMF) profiles describing the dissociation between each ligand and PARP1/PARP2 under physiological NaCl concentration (150 mM) and room conditions, in explicit solvent and ions. The center-of-mass (COM) distance between the protein binding pocket and the ligand is used as the reaction coordinate. Curves are shown for saruparib (A) and veliparib (B). The statistical uncertainty is represented as color shadowing corresponding to the standard deviation obtained from trajectory segments. Fig B Comparison between experimental and simulated binding free energy differences. Experimental (Δ(ΔGExperimental)PARP1−PARP2) and simulated (Δ(ΔGPMF Simulation)PARP1−PARP2) binding free energy differences for different inhibitors (saruparib, NMS-P118, veliparib, and olaparib). A linear shading is included as a guide to the eye. PARP1-selective inhibitors are represented as stars, whereas non-selective inhibitors are shown as circles. Fig C Intermolecular contact frequency maps for PARP1 and PARP2. Contact frequency maps between protein residues and ligand heavy atoms. Panels (A–D) correspond to PARP1 in complex with NMS-P118, saruparib, olaparib, and veliparib, respectively. Panels (E–H) correspond to the same ligands in complex with PARP2. Contacts are represented as percentage frequencies. Fig D Binding site interaction energies for PARP2–ligand complexes. Per-residue binding free energy decomposition mapped onto the protein surface for NMS-P118 (A), olaparib (B), saruparib (C), and veliparib (D). Left panels show total contributions, while right panels display the decomposition into van der Waals, electrostatic, polar solvation, and non-polar solvation components. Only residues with contributions larger than 1 kcal/mol are shown. Fig E MM-GBSA binding free energy profiles for PARP1 complexes. (A) Binding free energy profiles along molecular dynamics trajectories for different inhibitors. (B–E) Per-residue energy decomposition for NMS-P118, olaparib, sarupar [file pcbi.1014296.s001.pdf]

S1 Appendix
Cooperative molecular interaction networks govern PARP1 inhibitor selectivity and binding affinity

Alejandro Feito

Department of Physical Chemistry, Universidad Complutense de Madrid, Madrid, Spain and Instituto Pluridisciplinar, Universidad Complutense de Madrid, Madrid, Spain

Natàlia DeMoya-Valenzuela, Cristian Privat, and Lucía Paniagua-Herranz

Experimental Therapeutics Unit, Hospital Clínico San Carlos (HCSC), Instituto de Investigación Sanitaria San Carlos (IdISSC), Madrid, Spain

Andrés R. Tejedor

Department of Physical Chemistry, Universidad Complutense de Madrid, Madrid, Spain
Yusuf Hamied Department of Chemistry, University of Cambridge, United Kingdom and Instituto Pluridisciplinar, Universidad Complutense de Madrid, Madrid, Spain

Adiran Garaizar

Data Science, Bayer AG, Monheim am Rhein, Germany

Alberto Ocana

Experimental Therapeutics Unit, Hospital Clínico San Carlos (HCSC), Instituto de Investigación Sanitaria San Carlos (IdISSC), Madrid, Spain and PhAsIca Biosciences S.L, Madrid, Spain

Jorge R. Espinosa

Department of Physical Chemistry, Universidad Complutense de Madrid, Madrid, Spain
Yusuf Hamied Department of Chemistry, University of Cambridge, United Kingdom
Instituto Pluridisciplinar, Universidad Complutense de Madrid, Madrid, Spain and PhAsIca Biosciences S.L, Madrid, Spain

(Dated: May 18, 2026)

SI. PREPARATION OF THE SYSTEMS AND SIMULATION DETAILS

All atomistic simulations were performed using the GROMACS simulation package (version 2023)[1]. Given our interest in exploring interaction potentials at physiological salt concentration (150 mM NaCl), with a solvent and ion model that accurately reproduces ion solubilities in water at 310 K, these configurations were then solvated in a cubic box (8.5 × 8.5 × 8.5 nm). Some water molecules were replaced by Na^+ and Cl^- ions to neutralize the system and achieve the desired salt concentration. Energy minimization was performed with a force tolerance of 1000 kJ mol^{-1} nm^{-1}. Bond constraints on hydrogen-containing bonds were enforced via the LINCS algorithm [2], allowing a time step of 2 fs. Periodic boundary conditions (PBC) were maintained, and electrostatics were computed using the Particle-Mesh Ewald (PME) [3] method with a Coulomb cutoff of 0.9 nm. Temperature coupling was performed using the velocity-rescale (v-rescale) thermostat with a relaxation time constant $\tau_T = 1.0$ ps, while pressure coupling was applied using the Parrinello–Rahman barostat with a relaxation time constant $\tau_P = 1.0$ ps.

For the potential-of-mean-force (PMF) simulations the configurations were solvated in a box of 8.5x8.5x15 nm at 300K and 150mM of NaCl. The center-of-mass (COM) distance between the protein and the ligand controlled using a harmonic umbrella potential with a pulling force constant of 10000 kJ mol^{-1} nm^{-2}. Approximately 60 umbrella sampling windows were defined, spaced every 0.025 nm within the range. For production runs, positional restraints of 1000 kJ mol^{-1} nm^{-2} (in directions perpendicular to the pulling axis) were applied to the amino acid heavy atoms of four residues of the protein with the most values of frequency contacts along the simulation with the ligand (to avoid rotations) and the heavy atoms of the drug were placed at the center of the molecule to allow freedom of movement of the regions that predominantly interact with the protein during the simulation. Each system was simulated for a total accumulated simulation time of 600 ns. The Weighted Histogram Analysis Method (WHAM) [4], as implemented in GROMACS, was used to reconstruct the free energy profiles. The initial 2000 ps of each simulation were excluded from WHAM analysis to ensure equilibration. Statistical errors of the PMF profiles were estimated by block analysis of the simulation trajectories. Each trajectory was divided into four independent segments, and PMF profiles were computed separately for each segment using the GROMACS WHAM tool. The statistical uncertainty at each point along

the reaction coordinate was then quantified as the standard deviation of the PMF values obtained from these four blocks. To further assess the convergence of the calculation, Figure S11 shows the PMF profiles obtained from the different accumulative trajectory segments for the PARP1–saruparib system.

SII. SEQUENCE OF PARP1 AND PARP2 AND PDB OF THE STRUCTURED DOMAINS AND SEQUENCE OF PARP1

PARP1:

MAESSDKLYRVEYAKSGRASCKKCSESIPKDSLRMAIMVQSPMFDGKVPHWYHFSCFWKVGHSIRHPDVEVDGFS
ELRWDDQQKVKKTAEAGGVTGKGQDGIGSKAEKTLGDFAAEYAKSNRSTCKGCMEKIEKGQVRLSKKMVDPEKPQ
LGMIDRWYHPGCFVKNREELGFRPEYSASQLKGFSLLATEDKEALKKQLPGVKSEGKRKGDEVDGVDEVAKKKSK
KEKDKDSKLEKALKAQNDLIWNIKDELKKVCSTNDLKELLIFNKQQVPSGESAILDRVADGMVFGALLPCEECSG
QLVFKSDAYYCTGDVTAWTKCMVKTQTPNRKEWVTPKEFREISYLKKLKVKKQDRIFPPETSASVAATPPPSTAS
APAAVNSSASADKPLSNMKILTLGKLSRNKDEVKAMIEKLGGKLTGTANKASLCISTKKEVEKMNKKMEEVKEAN
IRVVSEDFLQDVSASTKSLQELFLAHILSPWGAEVKAEPVEVVAPRGKSGAALSKKSKGQVKEEGINKSEKRMKL
TLKGGAAVDPDSGLEHSAHVLEKGGKVFSATLGLVDIVKGTNSYYKLQLLEDDKENRYWIFRSWGRVGTVIGSNK
LEQMPSKEDAIEHFMKLYEEKTGNAWHSKNFTKYPKKFYPLEIDYGQDEEAVKKLTVNPGTKSKLPKPVQDLIKM
IFDVESMKKAMVEYEIDLQKMPLGKLSKRQIQAAYSILSEVQQAVSQGSSDSQILDLSNRFYTLIPHDFGMKKPP
LLNNADSVQAKVEMLDNLLDIEVAYSLLRGGSDDSSKDPIDVNYEKLKTDIKVVDRDSEEAEIIRKYVKNTHATT
HNAYDLEVIDIFKIEREGECQRYKPFKQLHNRRLLWHGSRTTNFAGILSQGLRIAPPEAPVTGYMFGKGIYFADM
VSKSANYCHTSQGDPIGLILLGEVALGNMYELKHASHISKLPKGKHSVKGLGKTTPDPSANISLDGVDVPLGTGI
SSGVNDTSLLYNEYIVYDIAQVNLKYLLKLKFNFKTSLW

PARP1 Active Site:

NNADSVQAKVEMLDNLLDIEVAYSLLRGGSDDSSKDPIDVNYEKLKTDIKVVDRDSEEAEIIRKYVKNTHATTHN
AYDLEVIDIFKIEREGECQRYKPFKQLHNRRLLWHGSRTTNFAGILSQGLRIAPPEAPVTGYMFGKGIYFADMVS
KSANYCHTSQGDPIGLILLGEVALGNMYELKHASHISKLPKGKHSVKGLGKTTPDPSANISLDGVDVPLGTGISS
GVNDTSLLYNEYIVYDIAQVNLKYLLKLKFNFKTSLW

The following Protein Data Bank (PDB) codes were used for the atomistic simulations: PARP1 with saruparib (9ETQ [5]), with olaparib (7KK4 [6]), with veliparib (7KK6 [6]) and with NMS-P118 (5A00 [7]). For the wild-type protein we used the AlphaFold [8] prediction (model AF-P09874-F1).

PARP2:

MAARRRRSTGGGRARALNESKRVNNGNTAPEDSSPAKKTRRCQRQESKKMPVAGGKANKDRTEDKQDGMPGRSWA
SKRVSESVKALLLKGKAPVDPECTAKVGKAHVYCEGNDVYDVMLNQTNLQFNNNKYYLIQLLEDDAQRNFSVWMR
WGRVGKMGQHSLVACSGNLNKAKEIFQKKFLDKTKNNWEDREKFEKVPGKYDMLQMDYATNTQDEEETKKEESLK
SPLKPESQLDLRVQELIKLICNVQAMEEMMMEMKYNTKKAPLGKLTVAQIKAGYQSLKKIEDCIRAGQHGRALME
ACNEFYTRIPHDFGLRTPPLIRTQKELSEKIQLLEALGDIEIAIKLVKTELQSPEHPLDQHYRNLHCALRPLDHE
SYEFKVISQYLQSTHAPTHSDYTMTLLDLFEVEKDGEKEAFREDLHNRMLLWHGSRMSNWVGILSHGLRIAPPEA
PITGYMFGKGIYFADMSSKSANYCFASRLKNTGLLLLSEVALGQCNELLEANPKAEGLLQGKHSTKGLGKMAPSS
AHFVTLNGSTVPLGPASDTGILNPDGYTLNYNEYIVYNPNQVRMRYLLKVQFNFLQLW

PARP2 Active Site:

TQKELSEKIQLLEALGDIEIAIKLVKTELQSPEHPLDQHYRNLHCALRPLDHESYEFKVISQYLQSTHAPTHSDY
TMTLLDLFEVEKDGEKEAFREDLHNRMLLWHGSRMSNWVGILSHGLRIAPPEAPITGYMFGKGIYFADMSSKSAN
YCFASRLKNTGLLLLSEVALGQCNELLEANPKAEGLLQGKHSTKGLGKMAPSSAHFVTLNGSTVPLGPASDTGIL
NPDGYTLNYNEYIVYNPNQVRMRYLLKVQFNFLQLW

The following Protein Data Bank (PDB) codes were used for the atomistic simulations: PARP2 with olaparib (4TVJ [6]), with veliparib (3KJD [9]) and with NMS-P118 (4ZZY [7]). For the wild-type protein we used the AlphaFold prediction (model AF-Q9UGN5-F1).

SIII. CALCULATING CONTACT MAPS

Computation of intermolecular and intramolecular contact maps within protein is calculated from all-atom trajectories. Typically, molecular contacts are identified based on a distance criterion, with the assumption that the relative frequency of contact map occurrences (rather than absolute frequency) remains generally unaffected by the selected cut-off distance used in calculations, assuming the cut-off values are reasonable. In particular, we use a criterion that depends on the specific excluded volume of each amino acid, adopting a sequence-dependent cut-off distance equivalent to $1.2\sigma_{ij}$, where σ_{ij} represents the mean excluded volume of the respective ith and jth amino acids [10]. For the heavy atoms of drug molecules, a σ value of 5 Å was assigned. Given that the minimum of the potential used lies at approximately $2^{1/6}\sigma_{ij} \approx 1.122\sigma_{ij}$, we set the cut-off distance slightly beyond this point, at $1.2\sigma_{ij}$, to ensure significant binding.

SIV. CALCULATING BINDING FREE ENERGY AND HYDROGEN BOND FORMATION

We estimated the binding free energy ($\Delta G_{binding}$) of the ligand-protein complexes using the MMPBSA.py module [11] from AmberTools23 [12] with the molecular mechanics generalized Born surface area (MMGBSA) approach. The binding free energy was calculated every 10 frames of the molecular dynamics simulation trajectory. The total $\Delta G_{binding}$ was decomposed into electrostatic, van der Waals (vdW), polar solvation, and non-polar solvation components (Eq. S11) through pairwise per-residue analysis (idecomp = 4).

$$\Delta G_{\text{binding}} = \Delta E_{\text{elec}} + \Delta E_{\text{vdW}} + \Delta G_{\text{polar}} + \Delta G_{\text{nonpolar}} - T\Delta S \tag{S1}$$

The electrostatic (ΔE_{elec}) and van der Waals (ΔE_{vdW}) terms were calculated directly from the molecular mechanics force field as the sum of pairwise atomic interactions. The polar solvation term (ΔG_{polar}) was computed using the modified generalized Born model developed by Onufriev, Bashford, and Case (igb=5) [13], while the non-polar contribution ($\Delta G_{nonpolar}$) was estimated using solvent-accessible surface area (SASA) default model (gbsa=2). Default parameters were applied in both solvation contributions. The entropic component ($T\Delta S$) was not included in the estimation of the binding free energy.

In addition, hydrogen bond formation was analyzed throughout the trajectories using CPPTRAJ [14], enabling the identification of persistent hydrogen-bonding interactions between the ligand and protein residues. A hydrogen bond is defined as an interaction involving an acceptor heavy atom (A), a donor hydrogen atom (H), and its corresponding donor heavy atom (D). The interaction was identified present when the distance between the acceptor and donor heavy atoms was less than 3.0 Å and the A–H–D angle was greater than 135°.

SV. CALCULATING ROOT MEAN SQUARE DEVIATION AND FLUCTUATION

We quantified the structural stability and flexibility of the PARP1 system by calculating the Root Mean Square Deviation (RMSD) and Root Mean Square Fluctuation (RMSF) from all-atom molecular dynamics trajectories using the package MDAnalysis in Python3.

The RMSD, which measures the average displacement of atoms relative to a reference

structure over time, was calculated for the protein's Cα atoms using the following formula:

$$\mathrm{RMSD}(t) = \sqrt{\frac{1}{N}\sum_{i=1}^{N}\left|r_i(t) - r_i^{\mathrm{ref}}\right|^2} \tag{S2}$$

where N is the total number of atoms considered (in this case, Cα atoms), $r_i(t)$ is the position vector of the i-th atom at time t, and r_i^{ref} is the position vector of the corresponding atom in the reference structure.

Subsequently, the RMSF was calculated to evaluate the per-residue flexibility. The RMSF for each residue α is defined as:

$$\mathrm{RMSF}(\alpha) = \sqrt{\frac{1}{T}\sum_{t=1}^{T}\left(r\alpha(t) - \langle r\alpha\rangle\right)^2} \tag{S3}$$

where T is the total number of frames in the trajectory, $\vec{r}\alpha(t)$ is the position vector of the Cα atom of residue α at time t, and $\langle\vec{r}\alpha\rangle$ is the time-averaged position of that same atom. For this analysis, the protein Cα atom was first aligned to the first configuration of the simulation.

To assess the effect of ligand binding on the conformational dynamics of PARP1 and PARP2, we computed RMSF and RMSD profiles for both apo proteins and their complexes with saruparib, NMS-P118, veliparib, and olaparib (Figure H).

For PARP1, the RMSF profile across residues 752–1002 (Figure HA) identifies regions of high intrinsic mobility, particularly near the α-helix F (residues 752–778). This segment shows marked flexibility in the apo form and reduced motion upon ligand binding, with saruparib, olaparib, and veliparib producing the most pronounced stabilization. The focused view of residues 852–902 (Figure HB) reveals that all ligands except NMS-P118 constrain the active-site loop region. The RMSD trajectories (Figure HC) indicate that saruparib yields the most stable complex, while NMS-P118 maintains apo-like deviations, and olaparib and veliparib reach intermediate stability levels.

For PARP2, the RMSF across residues 323–573 (Figure HD) shows a generally more rigid catalytic domain compared to PARP1, with localized ligand-dependent responses at the α-helix F (residues 323–348). Saruparib and veliparib reduce fluctuations relative to the apo state, whereas olaparib increases them. The zoomed-in region (residues 423–473, Figure HE) confirms this stabilizing effect for saruparib and veliparib. RMSD traces (Figure HF) show that PARP2 complexes with saruparib and veliparib achieve stable low-RMSD plateaus,

while olaparib and NMS-P118 exhibit trajectories similar to the apo protein, indicating less conformational restraint.

Structural representations of PARP1 in the apo form and bound to saruparib or NMS-P118 (Figures HG–I) illustrate these dynamic differences. Residue-level color mapping according to RMSF highlights the stabilization of helix F and the catalytic loop upon saruparib binding, while NMS-P118 allows these regions to remain partially flexible.

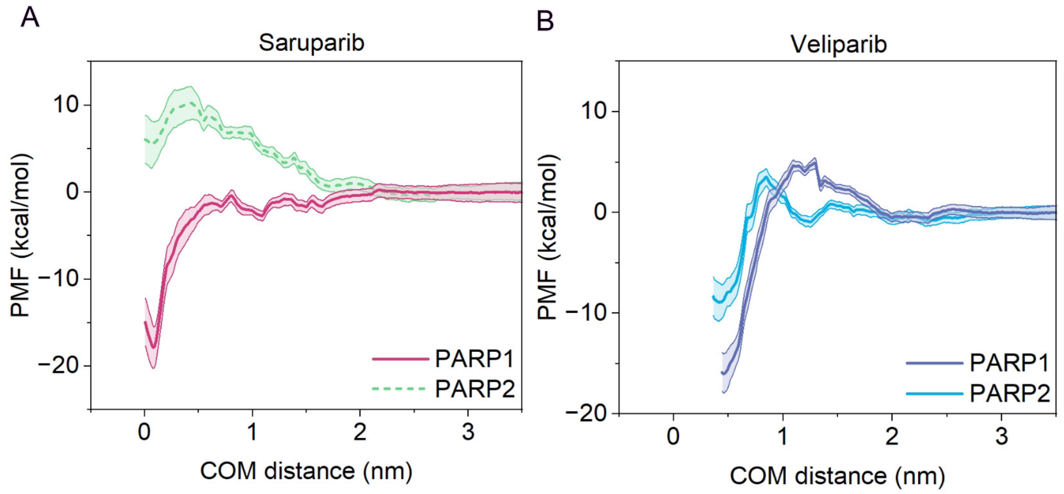

FIG A: Atomistic PMF dissociation profiles between each ligand and PARP1/PARP2 under physiological NaCl concentration (150 mM) and room conditions, in explicit solvent and ions. The COM distance between the protein binding pocket and the ligand is used as the reaction coordinate. Curves are shown for saruparib (A) and veliparib (B), depicting the statistical uncertainty of each PMF profile by a color shadowing corresponding to the standard deviation obtained from trajectory segments (see Section SI for further details).

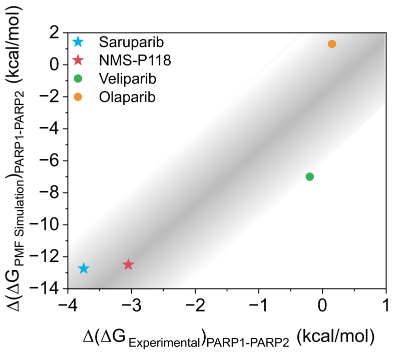

FIG B: Comparison between experimental ($\Delta(\Delta G_{\mathrm{Experimental}})_{\mathrm{PARP1-PARP2}}$) and simulated ($\Delta(\Delta G_{\mathrm{PMF\ Simulation}})_{\mathrm{PARP1-PARP2}}$) binding free energies for different inhibitors (Saruparib, NMS-P118, Veliparib, and Olaparib) for PARP1 and PARP2. A linear shading to the obtained correlation was added as a guide to the eye. Specific inhibitors of PARP1 are plotted in stars and non-specific inhibitors in circles.

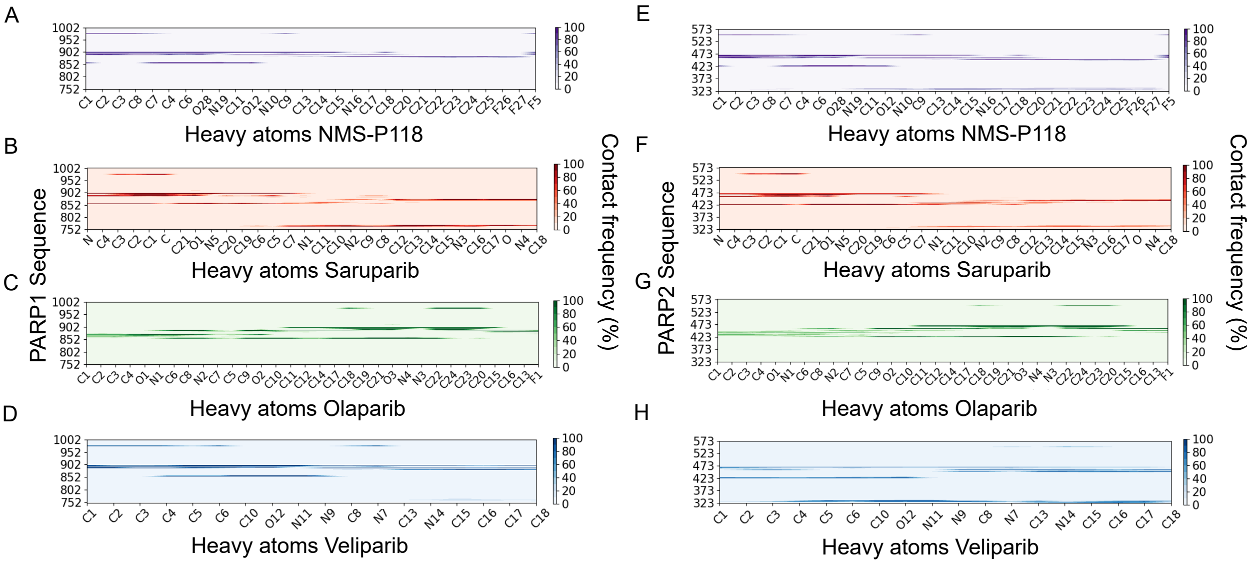

FIG C: Intermolecular frequency contact maps for PARP1 protein with heavy atoms of NMS-P118 (A), saruparib (B), olaparib (C) and veliparib (D). Intermolecular frequency contact maps for PARP2 protein with heavy atoms of NMS-P118 (E), saruparib (F), olaparib (G) and veliparib (H). The contacts are represented as contact frequency in percentage.

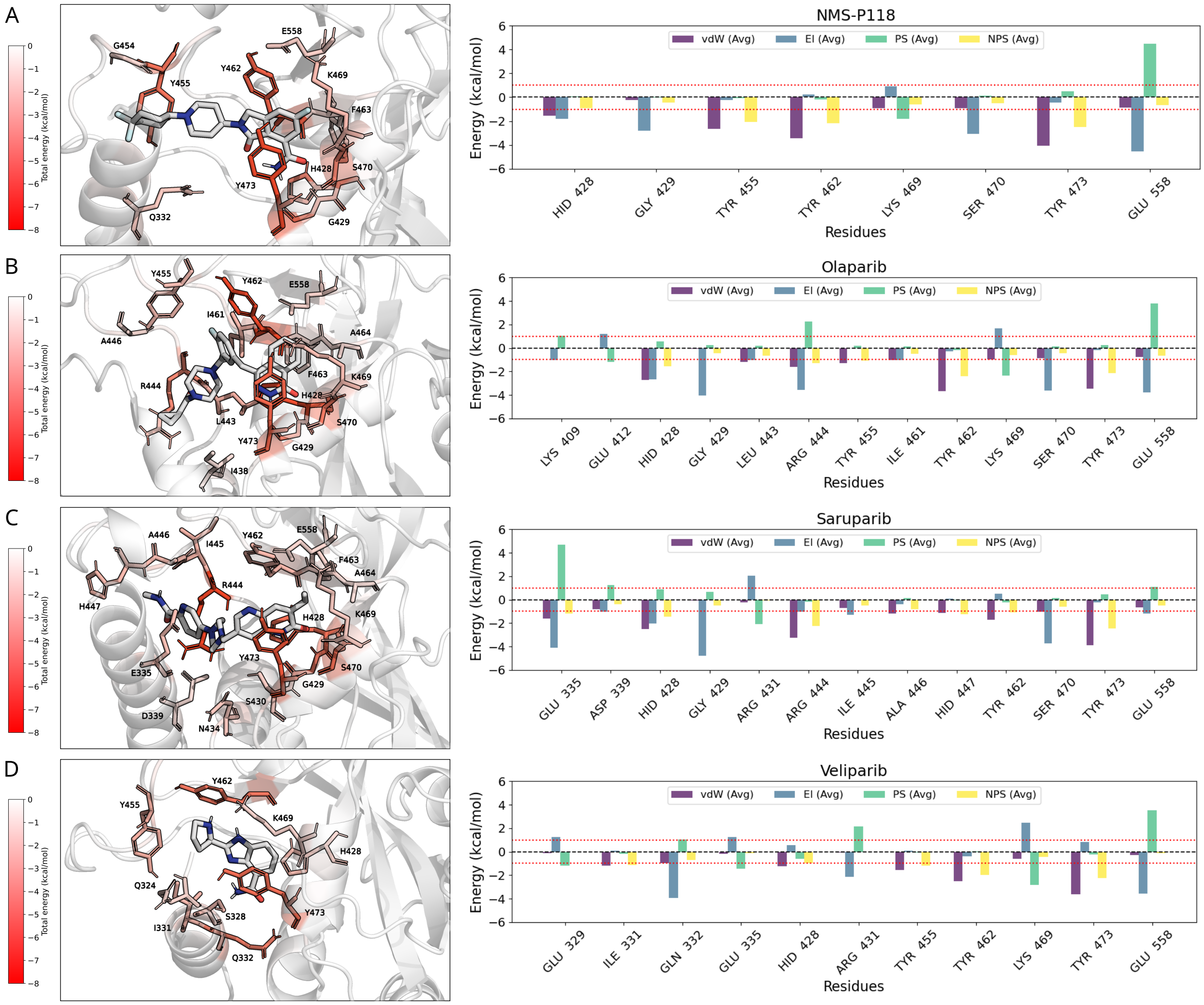

FIG D: Binding site interaction energies for PARP2 with NMS-P118 (A), olaparib (B), saruparib (C), and veliparib (D). Left panels: total per-residue binding free energy decomposition mapped onto the protein surface, colored from low (white) to high (red) contribution (kcal/mol). Right panels: Average energy of the protein-ligand interactions between PARP1 and different inhibitors. Only residues with an absolute energy contribution greater than 1 kcal/mol are shown. Energy components are broken down into van der Waals forces (vdW, blue), electrostatic interactions (El, purple), solvent polarization (PS, green), and non-polar solvent contribution (NPS, yellow).

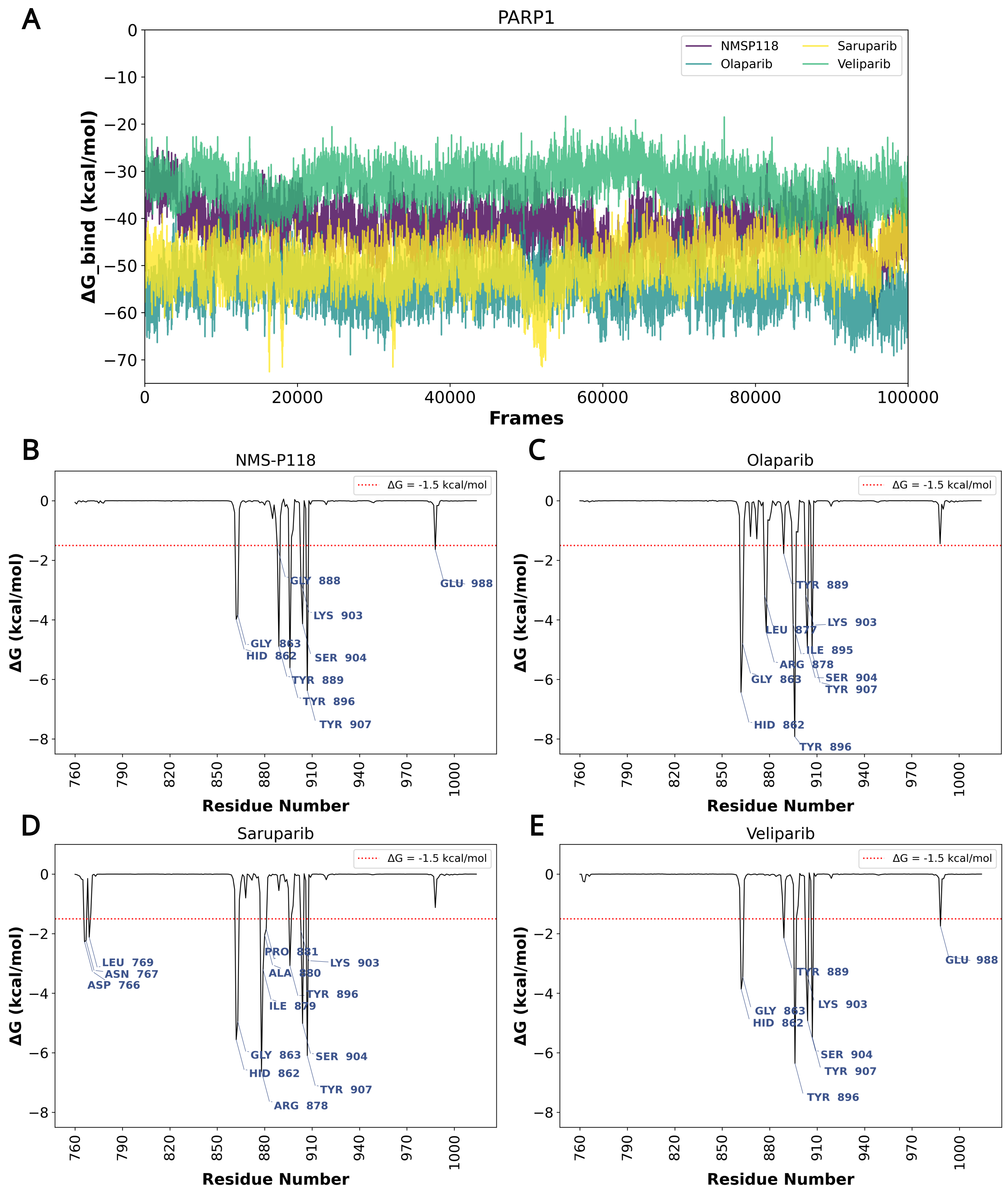

FIG E: (A) Binding free energy profiles calculated by MM-GBSA for NMS-P118, olaparib, saruparib, and veliparib in complex with PARP1 throughout the molecular dynamics trajectories. (B–E) Per-residue energy decomposition for NMS-P118 (B), olaparib (C), saruparib (D), and veliparib (E), highlighting the key residues that contribute most significantly to ligand binding. The red dashed line indicates the -1.5 kcal/mol threshold used to identify relevant interactions.

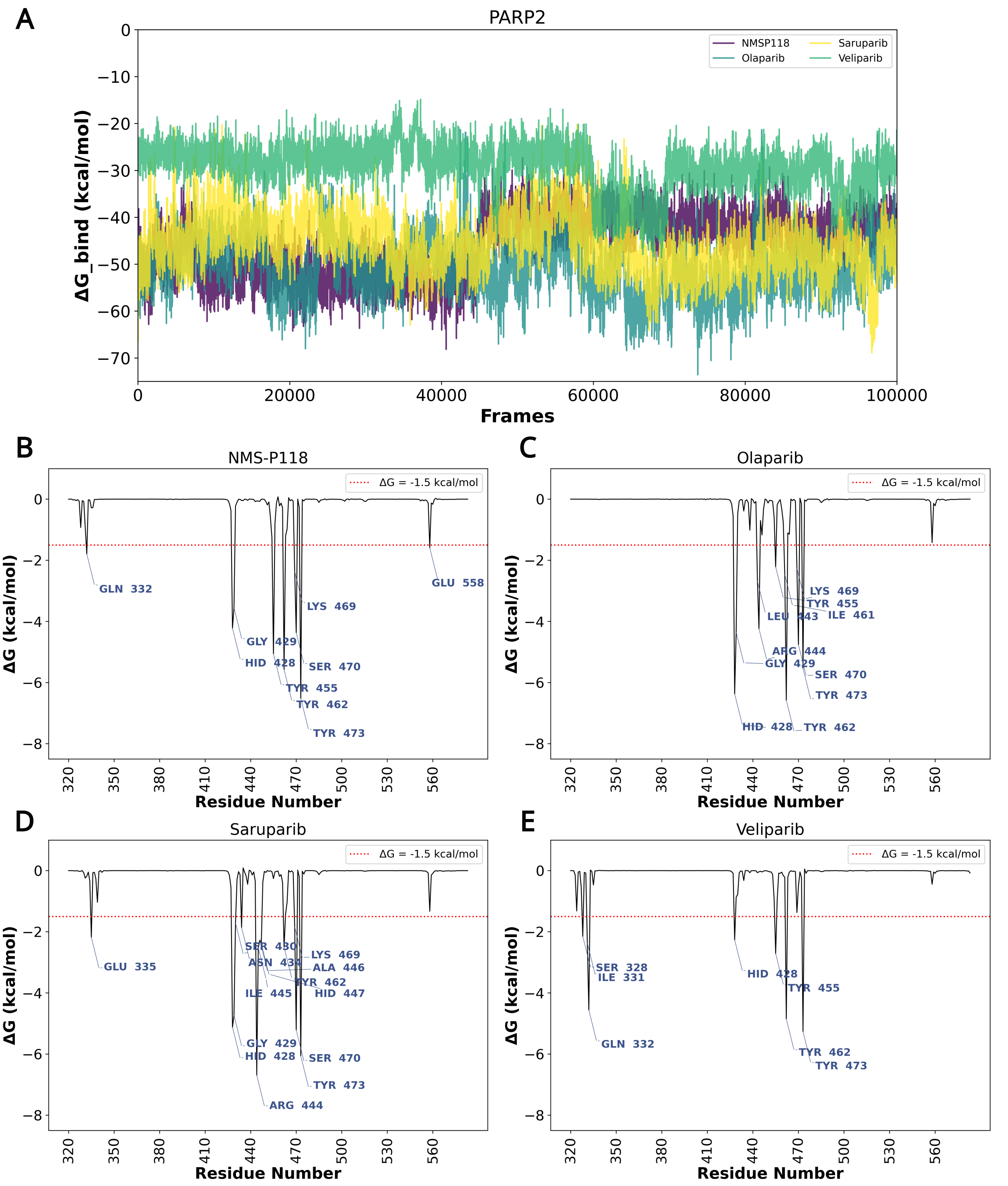

FIG F: (A) Binding free energy profiles calculated by MM-GBSA for NMS-P118, olaparib, saruparib, and veliparib in complex with PARP2 throughout the molecular dynamics trajectories. (B–E) Per-residue energy decomposition for NMS-P118 (B), olaparib (C), saruparib (D), and veliparib (E), highlighting the key residues that contribute most significantly to ligand binding. The red dashed line indicates the -1.5 kcal/mol threshold used to identify relevant interactions.

		PARP1						PARP2					
**Ligand**	**AA**	**Residue**	**Donor**	**Acceptor**	**Distance**	**Angle**	**Frequency**	**Residue**	**Donor**	**Acceptor**	**Distance**	**Angle**	**Frequency**
**NMS-P118**	SER	904	O(P)	O(L)	2,80	163,19	54%	470	O(P)	O(L)	2,81	163,91	58%
	GLY	863	N(P)	O(L)	2,89	162,62	54%	429	N(L)	O(P)	2,87	155,37	55%
			-	-	-	-	-		N(P)	O(L)	2,89	156,47	50%
**Olaparib**	GLY	863	N(L)	O(P)	2,83	162,82	86%	429	N(L)	O(P)	2,86	161,50	73%
			N(P)	O(L)	2,88	162,43	73%		N(P)	O(L)	2,86	155,01	71%
	SER	904	O(P)	O(L)	2,80	163,46	70%	470	O(P)	O(L)	2,80	165,87	71%
	TYR	896	N(P)	O(L)	2,89	163,97	56%	-	-	-	-	-	-
**Saruparib**	GLY	863	N(P)	O(L)	2,86	162,08	77%	429	N(L)	O(P)	2,83	162,22	87%
			N(L)	O(P)	2,86	161,51	63%		N(P)	O(L)	2,88	158,94	62%
	SER	904	O(P)	O(L)	2,81	162,28	58%	470	O(P)	O(L)	2,79	163,16	65%
	ILE	879	N(P)	O(L)	2,85	151,26	58%	-	-	-	-	-	-
**Veliparib**	SER	904	O(P)	O(L)	2,83	164,38	65%	-	-	-	-	-	-

FIG G: Summary of hydrogen bonds detected between PARP1 or PARP2 and different inhibitors (NMS-P118, olaparib, saruparib, veliparib) in molecular dynamics simulations. Only interactions with an occupancy above 50% are shown.

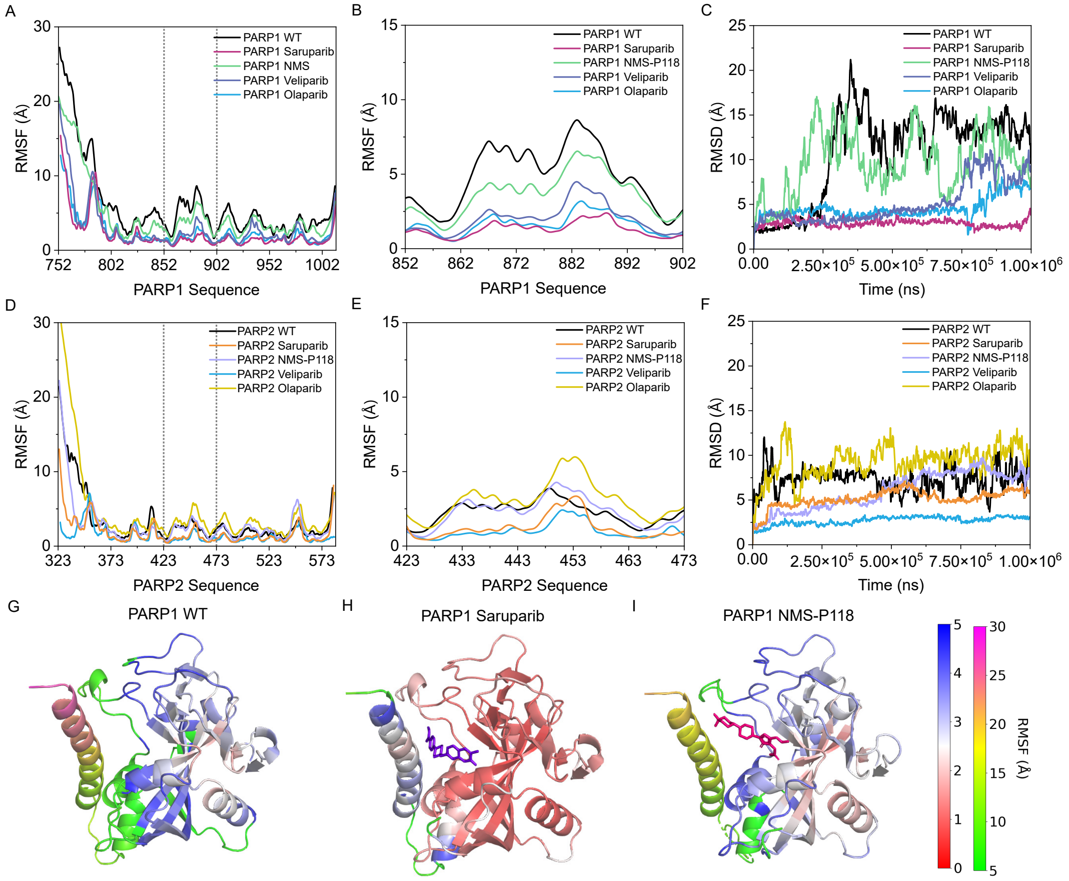

FIG H: Structural dynamics of PARP1 and PARP2 in the apo form and in complex with different inhibitors. Root Mean Square Fluctuation (RMSF) profiles of PARP1 for the wild-type (WT) protein and in complex with saruparib, NMS-P118, veliparib, and olaparib. (A) Global RMSF across residues 752–1002; (B) zoomed-in view of the flexible region spanning residues 852–902; (C) time evolution of the Root Mean Square Deviation (RMSD) for each system. Equivalent analyses for PARP2. (D) Global RMSF across residues 323–573; (E) zoomed-in view of residues 423–473; (F) RMSD over time for the different complexes and WT. Structural representations of PARP1 in the apo form (G), in complex with saruparib (H), and in complex with NMS-P118 (I). Proteins are shown in cartoon representation and colored according to per-residue RMSF values (see color bar), highlighting flexible regions and drug-induced stabilization effects.

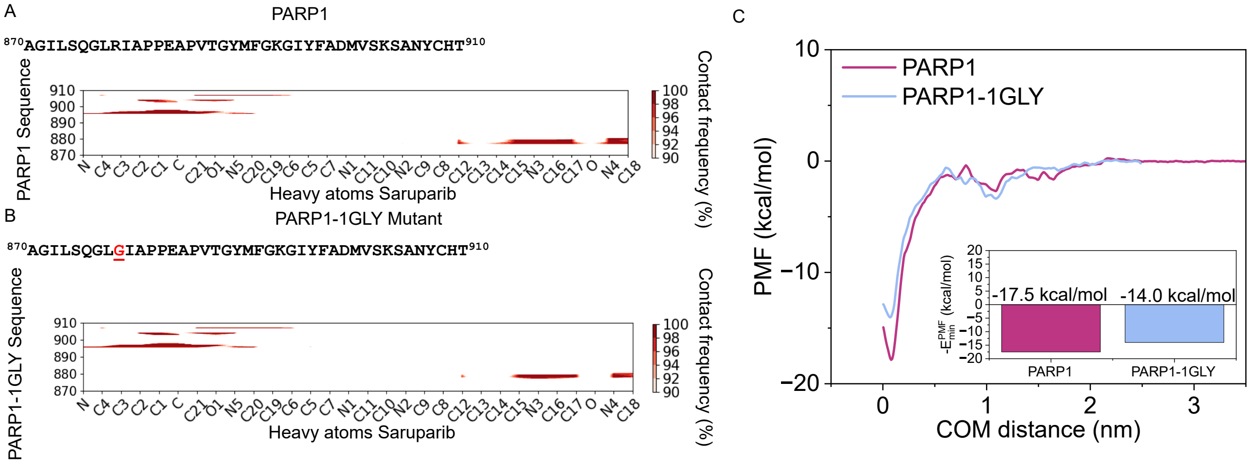

FIG I: Residue–ligand contact frequency maps between saruparib and PARP1 (A) and the 1GLY mutant (B). The PARP1 sequence is shown above each map, with the glycine substitution highlighted in red. Contact frequencies are calculated between heavy atoms of saruparib and zoom in the residues 870–910 of PARP1. The contacts are represented as contact frequency in percentage. (C) Potential of Mean Force (PMF) profiles for the unbinding of saruparib from PARP1 and the 1GLY mutant, computed as a function of the center-of-mass (COM) distance between the ligand and the protein. The inset shows the minimum binding free energy values derived from the PMF curves.

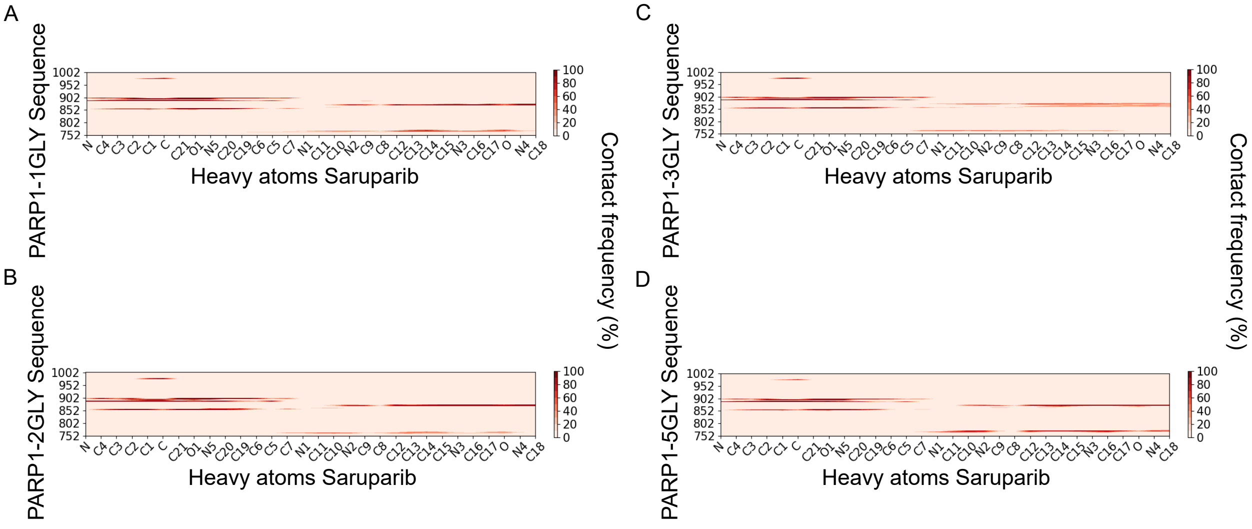

FIG J: Residue–ligand contact frequency maps between saruparib and 1GLY mutant (B), 2GLY mutant (B), 3GLY mutant (C) and 5GLY mutant (D). The contacts are represented as contact frequency in percentage.

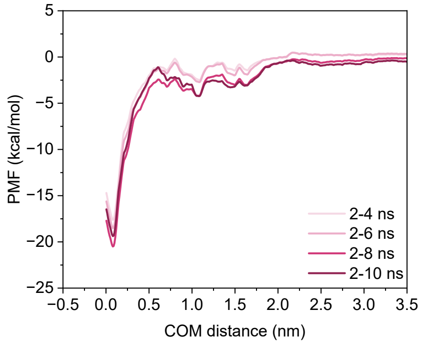

FIG K: Convergence analysis of the PMF profile for the PARP1–saruparib complex. The PMF curves were calculated using different acummulative trajectory segments (2–4 ns, 2–6 ns, 2–8 ns, and 2–10 ns).

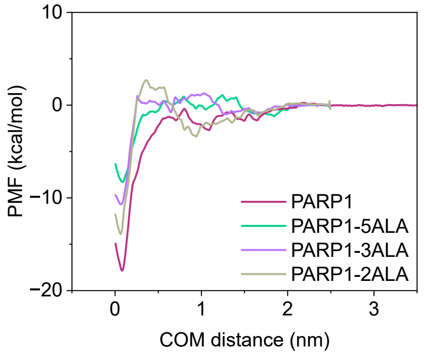

FIG L: PMF profiles for saruparib-PARP1 complex and the alanine mutants (PARP1-2ALA, PARP1-3ALA, and PARP1-5ALA), computed as a function of the center-of-mass distance between the ligand and the protein binding pocket.

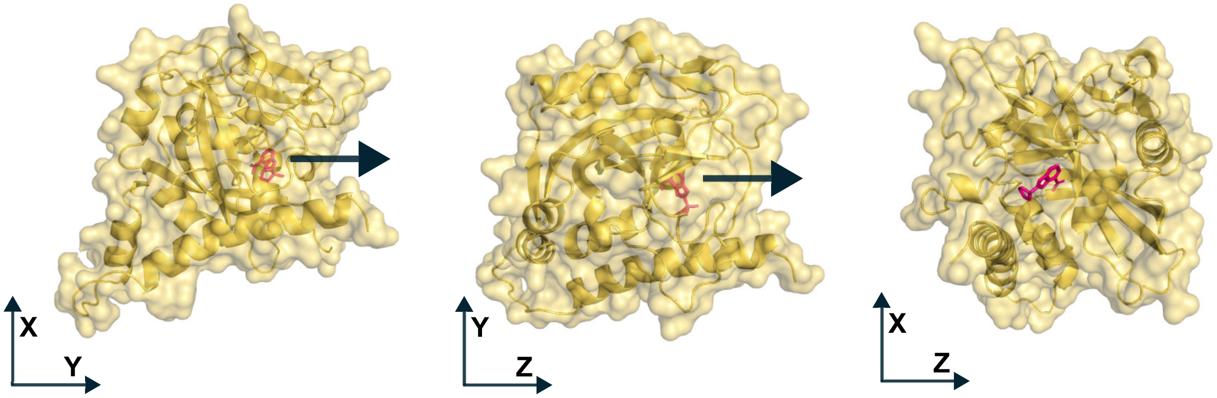

FIG M: Three orthogonal views of the protein–ligand complex showing the chosen reaction coordinate for PMF calculations. The arrows indicate the center-of-mass (COM) distance along which the ligand (veliparib) unbinding was sampled.

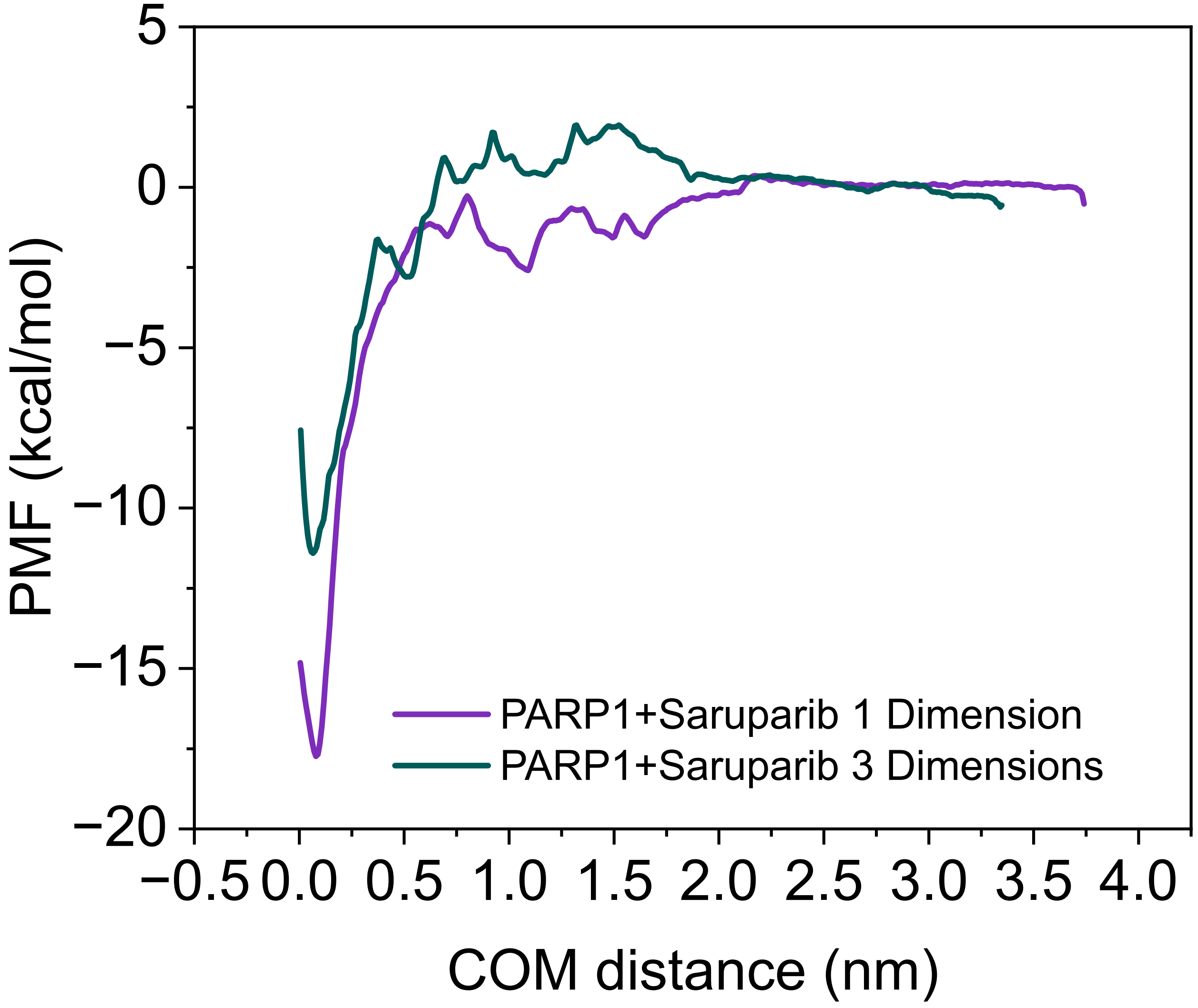

FIG N: Comparison of the potential of mean force (PMF) profiles for the PARP1–saruparib complex calculated using different dimensional descriptions of the dissociation pathway. The violet curve corresponds to the one-dimensional PMF obtained along a single reaction coordinate whereas the green surface/profile represents the three-dimensional PMF calculation.

[1] M. J. Abraham, T. Murtola, R. Schulz, S. Páll, J. C. Smith, B. Hess, and E. Lindahl, Gromacs: High performance molecular simulations through multi-level parallelism from laptops to supercomputers, SoftwareX **1**, 19 (2015).

[2] S. Thallmair, M. Javanainen, B. Fábián, H. Martinez-Seara, and S. J. Marrink, Nonconverged constraints cause artificial temperature gradients in lipid bilayer simulations, The Journal of Physical Chemistry B **125**, 9537 (2021).

[3] U. Essmann, L. Perera, M. L. Berkowitz, T. Darden, H. Lee, and L. G. Pedersen, A smooth particle mesh ewald method, The Journal of chemical physics **103**, 8577 (1995).

[4] S. Kumar, J. M. Rosenberg, D. Bouzida, R. H. Swendsen, and P. A. Kollman, The weighted histogram analysis method for free-energy calculations on biomolecules. i. the method, Journal of Computational Chemistry **13**, 1011 (1992).

[5] J. W. Johannes, A. Y. Balazs, D. Barratt, M. Bista, M. D. Chuba, S. Cosulich, S. E. Critchlow, S. L. Degorce, P. Di Fruscia, S. D. Edmondson, *et al.*, Discovery of 6-fluoro-5-{4-[(5-fluoro-2-methyl-3-oxo-3, 4-dihydroquinoxalin-6-yl) methyl] piperazin-1-yl}-n-methylpyridine-2-carboxamide (azd9574): A cns-penetrant, parp1-selective inhibitor, Journal of Medicinal Chemistry **67**, 21717 (2024).

[6] K. Ryan, B. Bolaños, M. Smith, P. B. Palde, P. D. Cuenca, T. L. VanArsdale, S. Niessen, L. Zhang, D. Behenna, M. A. Ornelas, *et al.*, Dissecting the molecular determinants of clinical parp1 inhibitor selectivity for tankyrase1, Journal of Biological Chemistry **296** (2021).

[7] G. Papeo, H. Posteri, D. Borghi, A. A. Busel, F. Caprera, E. Casale, M. Ciomei, A. Cirla, E. Corti, M. D'Anello, *et al.*, Discovery of 2-[1-(4, 4-difluorocyclohexyl) piperidin-4-yl]-6-fluoro-3-oxo-2, 3-dihydro-1 h-isoindole-4-carboxamide (nms-p118): a potent, orally available, and highly selective parp-1 inhibitor for cancer therapy, Journal of medicinal chemistry **58**, 6875 (2015).

[8] J. Jumper, R. Evans, A. Pritzel, T. Green, M. Figurnov, O. Ronneberger, K. Tunyasuvunakool, R. Bates, A. Žídek, A. Potapenko, *et al.*, Highly accurate protein structure prediction with alphafold, nature **596**, 583 (2021).

[9] T. Karlberg, M. Hammarstrom, P. Schutz, L. Svensson, and H. Schuler, Crystal structure of the catalytic domain of human parp2 in complex with parp inhibitor abt-888, Biochemistry

49, 1056 (2010).

[10] A. R. Tejedor, A. Garaizar, J. Ramírez, and J. R. Espinosa, ‘rna modulation of transport properties and stability in phase-separated condensates, Biophysical Journal **120**, 5169 (2021).

[11] B. R. I. Miller, T. D. J. McGee, J. M. Swails, N. Homeyer, H. Gohlke, and A. E. Roitberg, Mmpbsa.py: An efficient program for end-state free energy calculations, Journal of Chemical Theory and Computation **8**, 3314 (2012).

[12] D. A. Case, H. M. Aktulga, K. Belfon, D. S. Cerutti, G. A. Cisneros, V. W. D. Cruzeiro, N. Forouzesh, T. J. Giese, A. W. Götz, H. Gohlke, S. Izadi, K. Kasavajhala, M. C. Kaymak, E. King, T. Kurtzman, T.-S. Lee, P. Li, J. Liu, T. Luchko, R. Luo, M. Manathunga, M. R. Machado, H. M. Nguyen, K. A. O'Hearn, A. V. Onufriev, F. Pan, S. Pantano, R. Qi, A. Rahnamoun, A. Risheh, S. Schott-Verdugo, A. Shajan, J. Swails, J. Wang, H. Wei, X. Wu, Y. Wu, S. Zhang, S. Zhao, Q. Zhu, T. E. I. Cheatham, D. R. Roe, A. Roitberg, C. Simmerling, D. M. York, M. C. Nagan, and K. M. J. Merz, Ambertools, Journal of Chemical Information and Modeling **63**, 6183 (2023).

[13] A. Onufriev, D. Bashford, and D. A. Case, Exploring protein native states and large-scale conformational changes with a modified generalized born model, Proteins: Structure, Function, and Bioinformatics **55**, 383 (2004), https://onlinelibrary.wiley.com/doi/pdf/10.1002/prot.20033.

[14] D. R. Roe and T. E. I. Cheatham, Ptraj and cpptraj: Software for processing and analysis of molecular dynamics trajectory data, Journal of Chemical Theory and Computation **9**, 3084 (2013).
